# Hepatitis B virus x protein induces epithelial-mesenchymal transition of hepatocellular carcinoma cells by regulating long non-coding RNA

**DOI:** 10.1186/s12985-017-0903-5

**Published:** 2017-12-19

**Authors:** Yinji Jin, Di Wu, Weiwei Yang, Mingjiao Weng, Yafei Li, Xuefei Wang, Xiao Zhang, Xiaoming Jin, Tianzhen Wang

**Affiliations:** 1 Department of Pathology, Harbin Medical University, Harbin, 150081 China; 2 Department of Obstetrics and Gynecology, First Affiliated Hospital of Harbin Medical University, Harbin, 150001 China

**Keywords:** Hepatocellular carcinoma, Hepatitis B virus x protein, Long non-coding RNA, Epithelial-mesenchymal transition, Metastasis

## Abstract

**Background:**

It has been widely accepted that hepatitis B virus X protein (HBx) plays an important role in hepatocellular carcinoma (HCC). This study aimed to explore the function of long non-coding RNAs (lncRNAs) in the epithelial-mesenchymal transition (EMT) induced by HBx.

**Methods:**

The association between HBx and EMT markers was detected using immunohistochemistry in HCC tissues. The effect of HBx on HCC EMT was assessed through morphological analysis, transwell assay, metastatic in vivo study and detection of EMT markers. LncRNA microarray was used to screen the differently expressed lncRNAs. Small interfering RNA and Western blot were used to analyse the function and mechanism of the locked lncRNA.

**Results:**

HBx was negatively correlated with the epithelial marker E-cadherin but positively correlated with the mesenchymal marker vimentin in HCC tissues. HBx induced the mesenchymal phenotype and improved the metastatic ability of HCC cells. Meanwhile, HBx down-regulated E-cadherin, whereas it up-regulated vimentin. In HCC cells, HBx altered the expression of 2002 lncRNAs by more than 2-fold. One of them was ZEB2-AS1. Inhibition of ZEB2-AS1 can compensate for the EMT phenotype and reverse the expression of EMT markers regulated by HBx. Additionally, HBx affected the Wnt signalling pathway.

**Conclusions:**

HBx promotes HCC cell metastasis by inducing EMT, which is at least partly mediated by lncRNAs.

## Background

Hepatocellular carcinoma (HCC) is one of the most common cancers with high mortality worldwide [1]. Hepatitis B virus (HBV) infection is regarded as the leading risk factor for the development of HCC [2]. The rate of metastasis and recurrence of HCC in patients with HBV infection is significantly higher than patients without HBV infection [3–5]. Most studies attribute the oncogenic function of HBV to the HBV x protein (HBx), which is encoded by HBV X gene and has been proven to promote HCC cells metastasis by regulating matrix metalloproteinases (MMPs), cell adhesion molecules, microRNAs (miRNAs), and so on [6, 7].

Epithelial-mesenchymal transition (EMT) is an important mechanism for cancer metastasis and recurrence [8]. The cancer cells undergoing EMT will acquire mesenchymal cell-like features and show some changes in morphology, molecular markers and metastatic ability [9]. Lee et al. found that HBx mediated the activation of STAT5b, which further improves cancer cell motility and invasiveness by inducing EMT in HCC [10]. Yang et al. reported that HBx induced EMT by activating c-Src in HCC cells [11]. Until now, some molecules have been proven to be involved in the regulation of EMT; however, the mechanism is still not well known.

In recent years, increasing evidence indicates that long non-coding RNAs (lncRNAs) play an important role in cancer development [12]. HBx has been identified to promote HCC proliferation and metastasis by regulating lncRNA expression [13, 14]. HULC is the first lncRNA with highly specific up-regulation in HCC [15]. Liu et al. found that single nucleotide polymorphisms (SNPs) in HULC decreased the susceptibility to HCC in HBV persistent carriers [16]. Anin vitro study indicated that HBx up-regulated HULC expression and then promoted HCC proliferation by suppressing p18 [17]. Subsequently, some lncRNAs were found to play a role in HCC development, including Dreh, LINE, and HOTAIR [18–20].

In this study, we determined the association between HBx and EMT in HCC tissues. Then, we detected the effect of HBx on the metastatic ability and EMT markers of HCC cells. Moreover, lncRNAs, which are regulated by HBx and mediate the oncogenic function of HBx, were determined in HCC cells. It is important for understand the mechanism of HCC metastasis and explore novel targets for treatment.

## Methods

### Tissue microarray

The tissue microarray was obtained from the Superchip Biotechnology Company in Shanghai. The microarray contains 47 HCC samples. None of the patients received any therapy prior to surgery, and all the patient samples had complete clinical information.

### Immunohistochemistry

Immunohistochemistry was performed on the tissue microarray slide conventionally. In brief, the slide was deparaffinized by xylene and rehydrated using a graded ethanol series. Then, 3% H_2_O_2_ was used to block endogenous peroxidase in the tissues. Antigen retrieval was completed using microwave treatment. Then, 5% bovine serum albumin was used to block nonspecific reactions.The slides were incubated with primary antibody against E-cadherin (SCBT, Santa Cruz, CA, USA, 1:100), vimentin (SCBT, 1:100) and HBx (US Biological, Swampscott, MA, USA, 1:100) at 4 °C overnight. The streptavidin-biotin peroxidase kit (ZSGB Bio, Beijing, China) was used to detect the bound antibodies. Finally, the band was visualized by DAB staining (ZSGB Bio).

The immunohistochemical result was scored using the intensity and extent. Staining intensity was quantified as follows: negative (0), weak (1), moderate (2) or strong (3). Staining extent was quantified according to the percentage of positive cells: none (0), <25% (1), 25–50% (2), 50–75% (3) or >75% (4). The final score was calculated as the intensity score multiplied by the extent score.

### Cell culture

The HCC cell line HepG2 and lentiviral vector packaging cell line 293TN were grown in Dulbecco’s modified Eagle medium (DMEM; HyClone, Logan, UT, USA) with 10% fetal bovine serum (FBS; Invitrogen, Carlsbad, CA, USA), 100 μg/ml streptomycin and 100 IU/ml penicillin at 37 °C in a humidified atmosphere containing 5% CO2.

### Immunocytochemistry

Cells were seeded in 24 well plates, where the slides were placed in advance. After 24 h, the slides were fixed with 10% formalin and permeabilized with 0.1% Triton X-100. Then, the slides were incubated with HBx, E-cadherin and vimentin antibodies at 37 °C for 2 h. The secondary antibodies with streptavidin-biotin peroxidase were used to detect the primary antibodies. Finally, the binding was visualized by DAB staining.

### Generation of stable cell line

The HBx overexpression lentiviral vector was constructed by inserting the synthetic HBx (genotype C) into CON238 vectors. The pseudo lentivirus was packaged according to a previous report [21]. To generate stable cells, HepG2 cells were incubated with 10 MOI of control lentiviral particles or HBx overexpression lentiviral particles for at least 12 h, then the GFP positive cells were sorted with a flow cytometry after 72 h of culture in normal medium.

### Transfection

Cells were seeded in 6 well plates, and the small interfering RNAs (siRNAs) (Invitrogen) of the LncRNAs were transfected into cells using Lipofectamine 2000 (Invitrogen) according to the manufacturer’s protocol. The scrambled sequence was used as a negative control (NC). The siRNA-ZEB2-AS1 sequence used was 5′-CAAAGGACACCTTTGGTTACCTGAA-3′ [22].

### Transwell assays

The cell migration and invasion assays were performed using transwell chambers with or without Matrigel (Becton Dickson, Bedford, MA, USA). Briefly, 2 × 10^4^ cells (HepG2-control and HepG2-HBx) suspended in serum-free media were plated in the upper chamber. DMEM media with 10% FBS was plated in the bottom chamber. The chamber was incubated at 37 °C for 24 h, and then the bottom of the chamber was stained with crystal violet. Cells were counted in five randomly selected 200× fields under light microscopy. The average cell number was used to reflect the invasive and migratory ability.

### Xenograft metastatic in vivo study

Twenty healthy BALB/c female nude mice (4–6 weeks old) were purchased from Beijing Vital River Laboratory Technology Co., Ltd. (China), and the experiments were conducted according to the applicable national and international guidelines. They were randomly divided into two groups receiving HepG2-control and HepG2-HBx cells, respectively. Then, 1 × 10^6^ cells were suspended in 100 μL of PBS and injected into the nude mouse via the tail vein. Eight weeks later, lung tissue was collected for detection of the number and volume of the metastatic tumours by pathological methods.

### Western blot

Protein was extracted from the cultured cells and was quantitated using a BCA assay (Beyotime, Haimen, China). Equal amounts of protein were separated by SDS-PAGE and transferred onto PVDF membranes. The membranes were incubated with anti-E-cadherin (1:500), anti-vimentin (1:500), anti-HBx (1:200), anti-ZEB2 (1:200) and anti-GAPDH (SCBT, 1:2000) at 4 °C overnight, and then they were incubated with the respective secondary antibody at 37 °C for 2 h. Resulting bands were detected by an ECL Western blotting detection system (Thermo Scientific, Rockford, IL, USA).

### RNA extraction and LncRNAs microarray

Total RNA was isolated from the cultured cells (HepG2-control and HepG2-HBx) using TRIzol reagent (Invitrogen). The A260/A280 was detected by a Nano Drop ND-2000 spectrophotometer and RNA bands separated by agarose gel electrophoresis were used to determine the quantity and quality of the RNA. A260/A280 ≥ 1.9 and clearly visible bands were confirmed in all the samples before further experiments. Microarray hybridization was performed by Shanghai Biotechnology Corporation using a Sure Print G3 human lncRNA 4 × 180 k V6 microarray (Agilent).

### Real time-PCR validation assay

The level of lncRNA was validated by real time PCR using a SYBR green PCR mix (TransGen Biotech, Beijing, China) and BIO-RAD CFX96™ Real-Time System. LncRNA levels were normalized to GAPHD. PCR conditions were as follows: denaturation at 95 °C for 5 min, 39 cycles of amplification at 95 °C for 10 s/cycle, and then 60 °C for 30 s. Melting curve analyses were performed on the PCR products by progressive heating from 65 °C to 95 °C. Primers (GenePharma Co., Shanghai, China) specific for the lncRNAs and GAPDH were as follows: ZEB2-AS1, forward (F) 5′-ATGAAGAAGCCGCGAAGTGT-3′ and reverse (R) 5′-CACACCCTAATACACATGCCCT-3′, and GAPDH, forward (F) 5′-ATGGGGAAGGTGAAGGTCG-3′ and reverse(R) 5′-GGGGTCATTGATGGCAACAATA-3’ [22, 23].

### Statistical analysis

All the data were presented as the mean ± SD. Student’s-test was used to compare the difference between the two groups. The relationship between the two groups was analysed by the Pearson correlation test. *P* < 0.05 was considered to indicate a statistically significant difference. The DAVID Bioinformatics Tool (https://david.ncifcrf.gov/, version 6.7) was used to complete the functional enrichment analysis of the gene ontology (GO) and Kyoto encyclopaedia of genes and genomes (KEGG) database. GO functional terms were limited to “Biological Process” and KEGG pathways with FDR < 0.05 were considered to be significant.

## Results

### HBx was associated with EMT in HCC

**Fig. 1:**
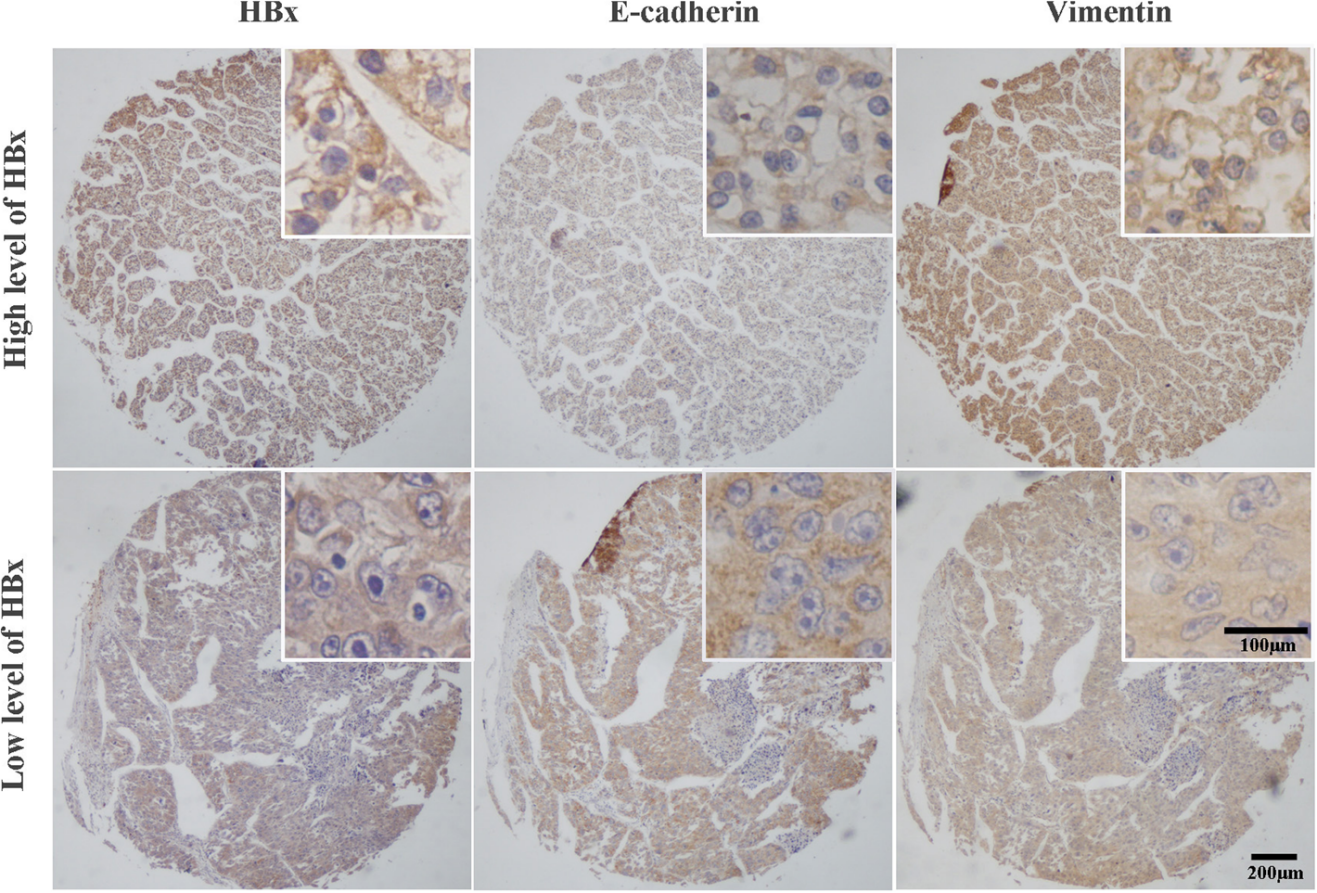
The expression of HBx and EMT markers was detected by immuno- histochemistry on the HCC tissue microarray. The expression of E-cadherin was decreased but that of vimentin was increased in the HCC tissues with high levels of HBx compared to those with low levels of HBx (100×).The top right corner is the enlarged image (400×)

To determine the association between HBx and EMT in HCC, we detected HBx and EMT markers in the HCC tissues by immunohistochemistry. Of 47 HCC samples, the level of E-cadherin was lower in the HCC tissues with HBx (33 cases) than those without HBx (14 cases), whereas the level of vimentin was on the opposite side (Table 1). HBx was negatively correlated with E-cadherin but positively correlated with vimentin (Fig. 1). The correlation coefficient was −0.65 between HBx and E-cadherin, while it was 0.70 between HBx and vimentin. This result indicated that HBx had an effect on EMT in HCC.

**Table 1:** The association between HBx and E-cadherin, vimentin and clinical parameters in HCC

Parameters	HBx negative (*n* = 14)	HBx positive (*n* = 33)	*P* Value
Age	50.0 ± 8.7	49.7 ± 9.3	
Sex (male/female)	13/1	27/6	
Grade			
I-II	8	21	
III-IV	6	12	
Tumour size (diameter)			
≤ 3 cm	7	14	
>3 cm	7	19	
AFP (≥300 ng/L)	6	11	
Creatinine (μmol/L)	68.0 ± 16.5	72.7 ± 40.5	
Urea nitrogen (mmol/L)	5.3 ± 0.6	5.2 ± 1.4	
CEA (ng/ml)	3.9 ± 2.3	3.1 ± 1.6	
E-cadherin	6.5 ± 2.5	4.1 ± 1.8	<0.05
Vimentin	3.5 ± 2.1	6.2 ± 3.1	<0.05

### HBx can promote HCC cell migration and invasion by regulating EMT

**Fig. 2:**
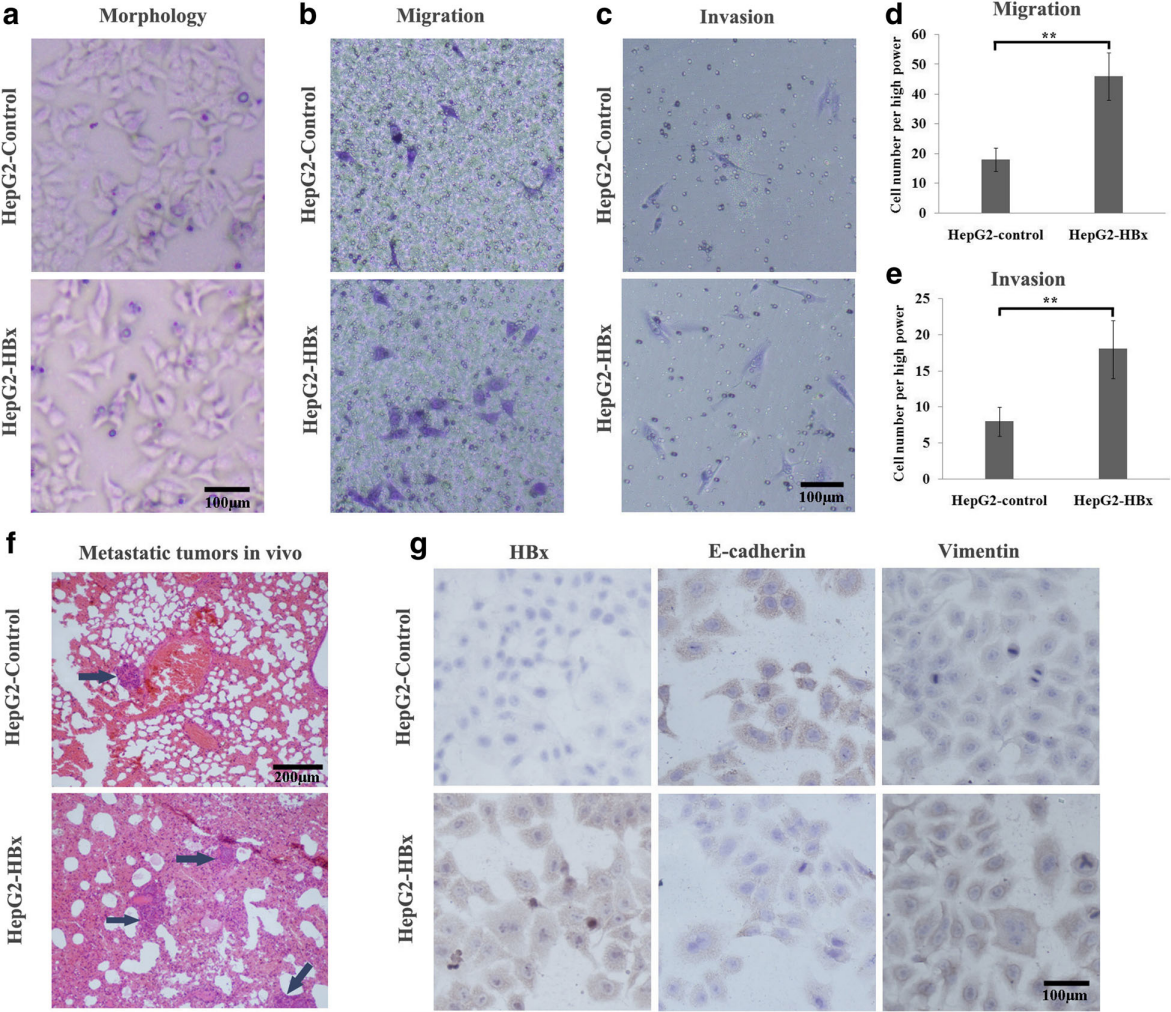
The effect of HBx on HCC EMT was detected by in vivo and in vitro experiments. **a** Morphology observation under the inverted microscope. HBx induced morphological alterations from the epithelial phenotype to the mesenchymal phenotype characterized by shuttle shape and scattered growth. **b** and **c** Transwell assays for migration and invasion. HBx promoted the metastatic ability and induced more cells to pass through the membrane. **d** and **e** The number of transmembrane cells are presented by column diagram. **f** Lung metastatic tumours in mice. The number of metastatic tumours was significantly higher in the HBx overexpressed HepG2 cells than the control group. **g** Immunocytochemistry assay for HBx, E-cadherin and vimentin. HBx overexpression in HepG2 cells resulted in decreased of E-cadherin and increased of vimentin. *p < 0.05, **p < 0.01

To identify the effect of HBx on EMT in HCC, we constructed an HBx-overexpressed HepG2 cell line by lentivirus infection and detected the cellular morphology, metastatic ability and expression of EMT markers. Under light microscopy, we found that HBx induced morphological change from the epithelial to mesenchymal phenotype (Fig. 2a). Transwell assays showed that HBx promoted the migratory and invasive ability of HepG2 cells. The number of cells passed through the wells was significantly higher in the HBx overexpressed HepG2 cells than the control cells (Fig. 2b-e). In vivo study showed that metastatic tumours formed in 70% (7/10) of the nude mice injected with the HepG2-control but in 100% (10/10) of those with HepG2-HBx. The number and size of the metastatic tumours also predominated in the HepG2-HBx group (Fig. 2f).The immunocytochemical result indicated that the epithelial marker of E-cadherin was down-regulated, whereas the mesenchymal marker of vimentin was up-regulated in the HBx overexpressed HepG2 cells (Fig. 2g). These data indicated that HBx might promote the metastatic ability of HCC cells by inducing EMT.

### HBx might regulate EMT by affecting the expression of lncRNAs in HCC

**Fig. 3:**
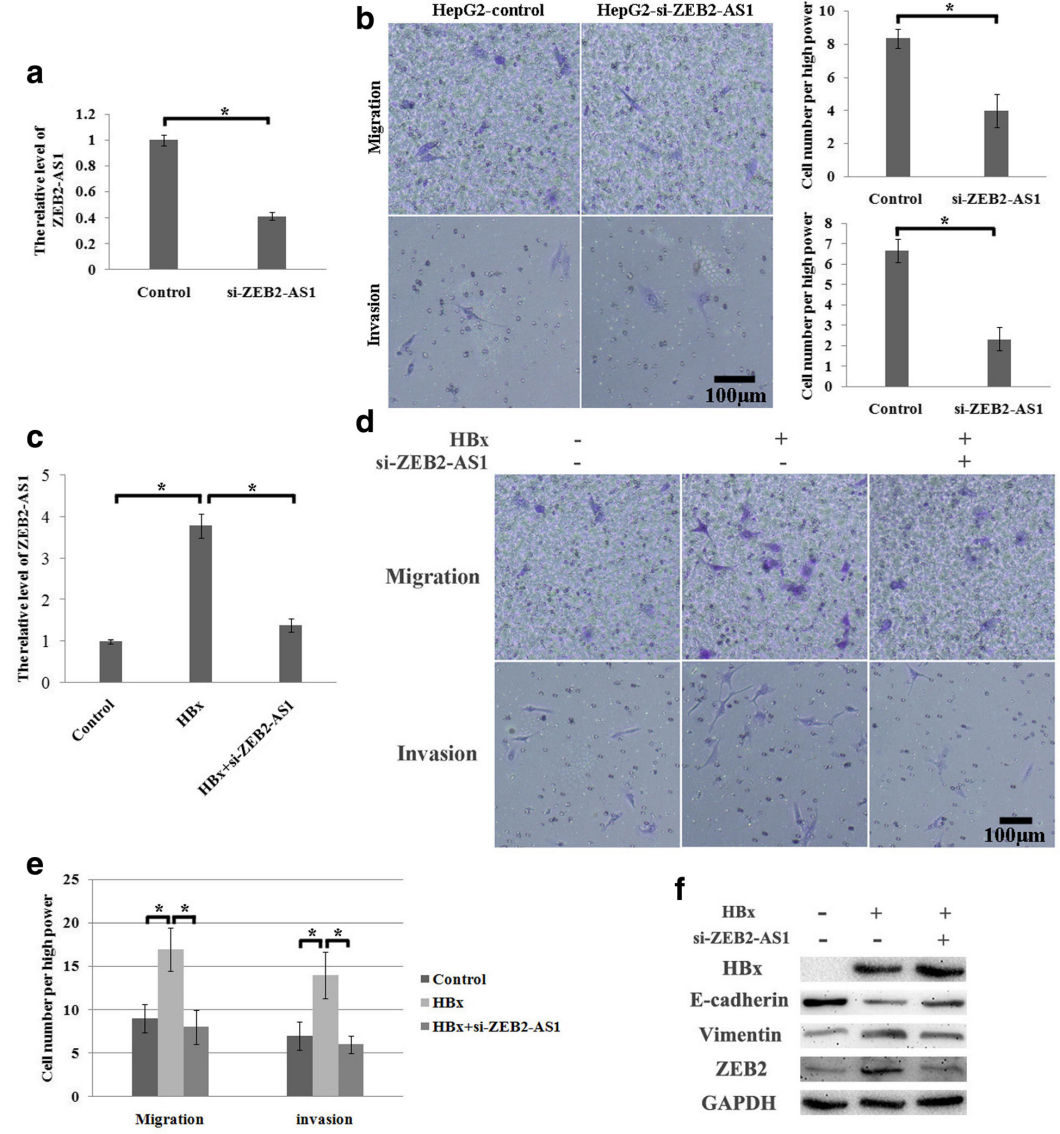
ZEB2-AS1 was an important lncRNA that mediated HBx-induced EMT. **a** The level of ZEB2-AS1 was verified by a real-time PCR assay. The siRNA can effectively inhibit the level of ZEB2-AS1. **b** Transwell assay. Knockdown of ZEB2-AS1 inhibited the metastatic ability of HepG2 cells. **c**. The level of ZEB2-AS1 was verified by a real-time PCR assay. ZEB2-AS1 was up-regulated by 3.79-fold by HBx, but compromised by si-ZEB2-AS1 in HepG2-HBx. **d** and **e** The regulating effect of ZEB2-AS1 on EMT was assessed by transwell assay. HBx promoted the metastatic ability of HCC cells; however, siRNA treatment against ZEB2-AS1 rescued the phenotype in these cells. **f** EMT markers were detected by Western blot. HBx overexpression enhanced vimentin and ZEB2 expression, whereas it inhibited E-cadherin expression. However, knockdown ZEB2-AS1 reversed the above protein expression. *p < 0.05, **p < 0.01

To explore the potential function of lncRNAs in HBx-regulated EMT, we compared the different expression of the lncRNAs between the HepG2-control and HepG2-HBx. The result indicated that 2002 LncRNAs were down-regulated or up-regulated by more than 2-fold in the HepG2 cells because of HBx. Of them, the ZEB2-AS1 (ZEB2-antisense RNA1), a known-EMT related lncRNA, was up-regulated by 3.55-fold. We first verified the function of ZEB2-AS1 in HepG2 cells. Knockdown of ZEB2-AS1 inhibited the metastatic ability of HepG2 cells (Fig. 3a and b). We further verified the level of ZEB2-AS1 by Q-PCR and that it was indeed increased in HepG2-HBx (Fig. 3c). Moreover, the metastatic ability was decreased in HepG2-HBx when ZEB2-AS1 was down-regulated by the siRNA technique (Fig. 3d and e). Western blot assays indicated that HBx up-regulated vimentin and down-regulated E-cadherin; however, knockdown of ZEB2-AS1 in HepG2-HBx reversed their expression (Fig. 3f). More interestingly, ZEB2, as one of the key regulatorsthat mediated the function of ZEB2-AS1, was also decreased with knockdown of ZEB2-AS1(Fig. 3f). That is, knockdown of ZEB2-AS1 can compromise the EMT process induced by HBx. Taken together, HBx might promote the EMT process by up-regulating the expression of ZEB2-AS1.

### HBx affected EMT associated genes expression in HCC

**Fig. 4:**
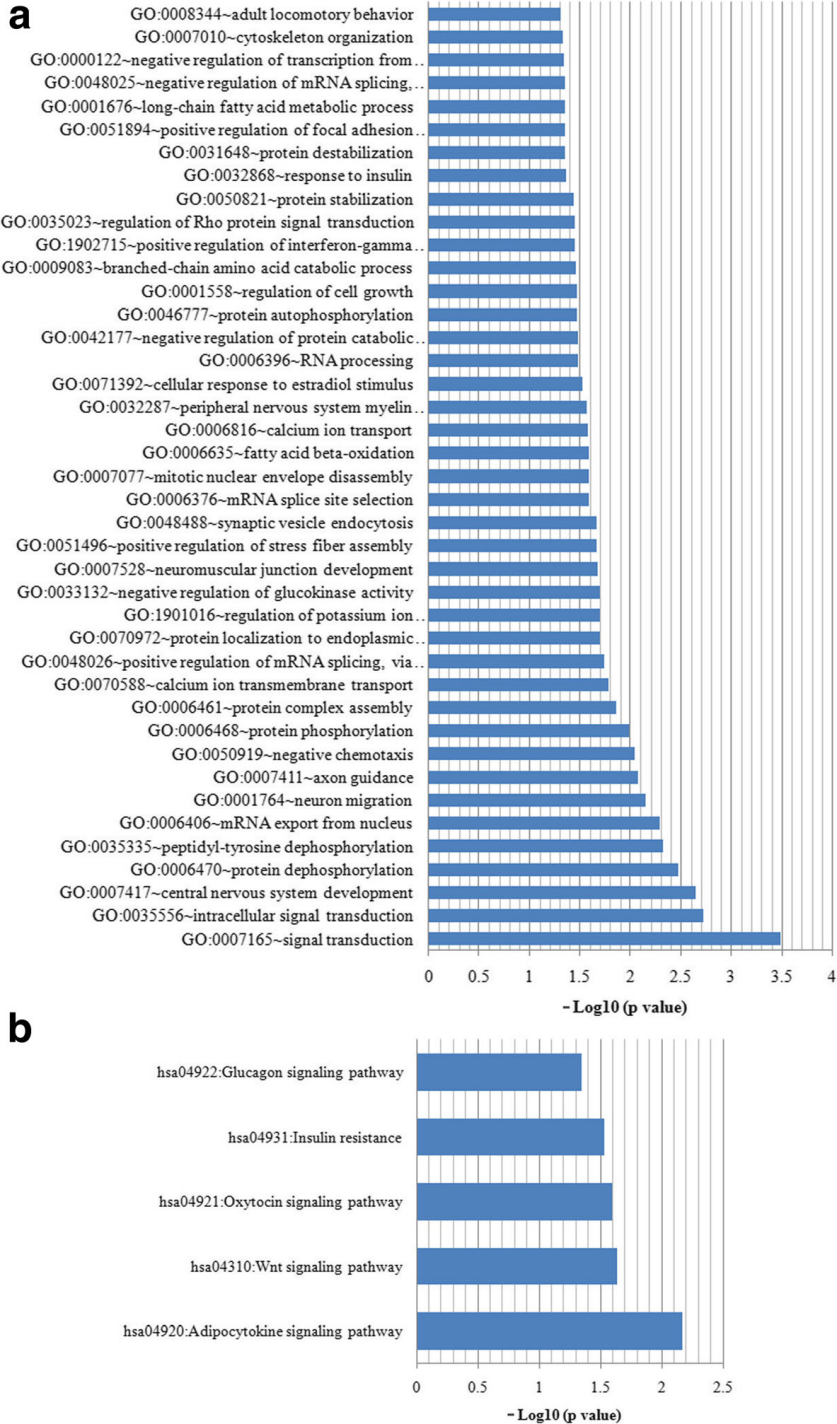
Analysis for microarray results. **a** GO enrichment analysis. All the altered mRNAs were significantly enriched in 41 GO terms. **b** KEGG pathway enrichment analysis. Five KEGG pathways were significantly enriched

Additionally, the microarray results also showed the different expression of mRNAs between the HepG2-control and HepG2-HBx. Two hundred sixty-two mRNAs were down-regulated and 197 mRNAs were up-regulated by HBx in the HepG2 cells. GO enrichment analysis revealed that all the altered mRNAs were significantly enriched in 41 GO terms (Fig. 4a), including cytoskeleton organization, and positive regulation of focal adhesion assembly. The KEGG pathway enrichment analysis indicated that five KEGG pathways were significantly enriched, in which the Wnt signalling pathway has been widely confirmed to play an important role in EMT (Fig. 4b). These results indicated that HBx regulated EMT associated genes expression.

## Discussion

HBx plays an important role during the development of HBV-associated HCC. Here, we verified that HBx can induce EMT in HCC. In 2006, Lee et al. first proved the effect of HBx on EMT. Their study found that HBx activated STAT5b, which further promoted cancer cell motility and invasiveness by inducing EMT [10]. Successively, the activation of c-Src, STAT3, Wnt, Akt and Notch1 were identified to mediate EMT induced by HBx [11, 24–27]. The importance of the Wnt signalling pathway was also presented by our result. In addition to the coding RNAs, HBx can also regulate lncRNA expression. Microarrays performed in mice and normal liver cells uncovered lots of lncRNAs altered by HBx [20, 28]. Recently, lncRNAs were found to mediate HBx-induced EMT. HBX-LINE1 is a hybrid RNA transcript of the human LINE1 and the HBV-encoded X gene, which promotes EMT-like changes by binding miR-122 [29]. Deng et al. found that HBx promoted EMT by enhancing the expression of Linc00152 in HCC [30]. Though we have these findings, we only know a little about the association between HBx and lncRNAs.

To analyse the function of lncRNAs in EMT induced by HBx, we compared the different expression of lncRNAs between HepG2-HBx and HepG2-control. The result indicated that multiple lncRNAs were altered. Of them, we have focused on ZEB2-AS1 in view of its association with EMT. ZEB2-AS1 is the natural antisense transcript corresponding to the 5’UTR of the zinc finger E-box binding homeobox 2. Over-expression of ZEB2-AS1 in epithelial cells can prevent the splicing of the Zeb2 5′-UTR and up-regulate the levels of ZEB2 protein (a transcriptional repressor of E-cadherin) [31]. Consequently, E-cadherin was decreased and resulted in EMT [31]. It was found that TGFβ1 from the cancer associated fibroblasts can induce EMT in the urinary bladder cancer cells by up-regulating ZEB2-AS1 and ZEB2 protein level [32]. ZEB2-AS1was also increased in the HCC tissues, and high levels of ZEB2-AS1 were biomarkers for poor prognosis [22]. Further study indicated that ZEB2-AS1 promoted HCC growth and metastasis by regulating ZEB2 and some EMT markers [22]. In this study, we proved that HBx induced EMT and up-regulated ZEB2-AS1 expression, whereas knockdown of ZEB2-AS1 compromised EMT in HBx overexpressed HepG2 cells. Therefore, ZEB2-AS1 mediated the occurrence of EMT induced by HBx in HCC cells. In addition to ZEB2-AS1, lots of lncRNAs were altered by HBx in HepG2 cells. These findings indicated that lncRNAs are involved in the development of HBV-associated HCC, though the functions of most of them are still not clear.

In this study, KEGG analysis divided the differentially expressed genes into five signalling pathways. Of them, the Wnt pathway is a well-known pathway associated with EMT, and the components of this pathway are involved in the EMT process [33, 34]. For example, β-catenin can translocate from the cytoplasm to the nucleus, where it regulates the expression of some genes associated with migration and invasion [35]. The activation of Wnt signalling can also up-regulate EMT-associated regulators, such as Snail and ZEB1 [36, 37]. Interestingly, it was reported that HBx can activate the Wnt signalling pathway by down-regulating the expression of the SFRPs (the antagonists of Wnt signalling pathway), thus promoting HBx-induced EMT [25]. Our result also indicated that HBX might promote EMT by activating the Wnt signalling pathway.

## Conclusions

In conclusion, this study verified that HBx can promote HCC development by inducing EMT. LncRNAs involved in HBx-induced EMT and ZEB2-AS1 were identified as key lncRNAs mediating this process. HBx might up-regulate ZEB2-AS1 and ZEB2, consequently inducing EMT. The Wnt signalling pathway was also associated with the occurrence of EMT induced by HBx. This study provided the evidence for the HBx-LncRNA-EMT axis. However, we do not have evidence with which to correlate ZEB2-AS1 and Wnt or other signalling pathways. In the complex network, there must be a certain link between the lncRNAs and all kinds of signalling pathways, which will be the goal of our explorations in the future.

## Abbreviations

DMEM: Dulbecco’s modified Eagle medium
EMT: Epithelial-mesenchymal transition
FBS: Fetal bovine serum
GO: Gene ontology
HBV: Hepatitis B virus
HBx: HBV x protein
HCC: Hepatocellular carcinoma
KEGG: Kyoto encyclopaedia of genes and genomes
lncRNAs: long non-coding RNAs
MMPs: Matrix metalloproteinases
NC: Negative control
SNPs: Single nucleotide polymorphisms
ZEB2: Zinc finger E-box binding homeobox 2
ZEB2-AS1: ZEB2-antisense RNA1
